# 162. Comparison of Clinical Risk Factors, Outcomes and Molecular Epidemiology of ESBL and non-ESBL Enterobacterales Bacteremia in Solid Organ Transplant Recipients

**DOI:** 10.1093/ofid/ofae631.048

**Published:** 2025-01-29

**Authors:** Zachary W Hanna, Navina K Birk, George J Alangaden, Geehan Suleyman, Jagjeet Kaur, Mayur Ramesh

**Affiliations:** Henry Ford Health, Detroit, MI; Henry Ford Hospital, Detroit, Michigan; Henry Ford Health, Detroit, MI; Henry Ford Health, Detroit, MI; Henry Ford Health, Detroit, MI; Henry Ford Hospital, Detroit, Michigan

## Abstract

**Background:**

Infections caused by extended-spectrum beta-lactamase (ESBL)-producing Enterobacterales are associated with adverse outcomes in recipients of solid organ transplants (SOTr). This study aims to elucidate the risk factors, outcomes, and molecular epidemiology associated with ESBL Enterobacterales bacteremia (ESBL EB) in abdominal SOTr.

**Table 1:**
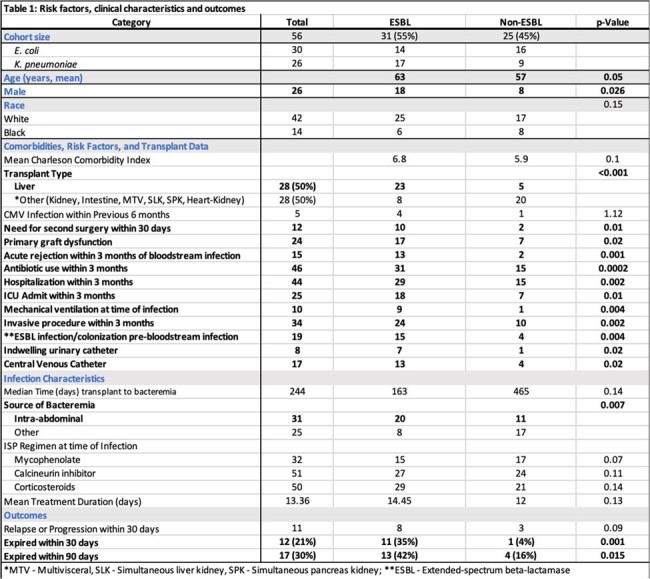
Risk Factors, Clinical Characteristics and Outcomes

**Methods:**

This observational cohort study was performed on abdominal SOTr with ESBL EB (cases) and non-ESBL EB (controls) at Henry Ford Health between August 2014 and February 2023. We performed whole genome sequencing on all blood isolates; sequencing libraries were created using +QIAGEN QIAseq FX DNA Library Kit according to manufacturer’s instructions and sequenced on Illumina NextSeq 2000. Fastq files were used to determine the distribution of traits and resistance genes using the 1928 Platform. Demographic, risk factors, clinical characteristics, outcomes and molecular sequence data were evaluated. Primary outcome was 30-day mortality.

**Figure 1:**
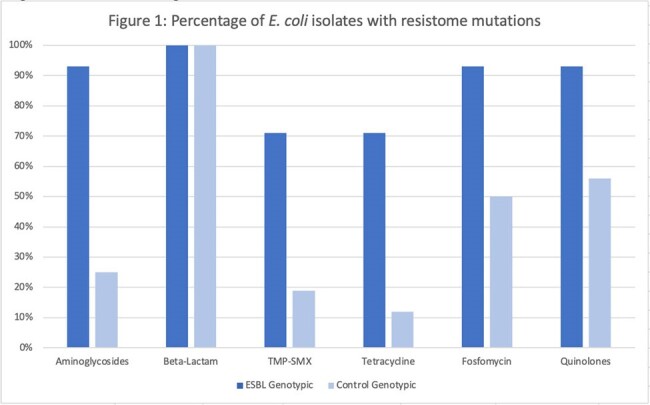
Percentage of E. coli isolates with resistome mutations

**Results:**

56 patients were included: 31 (55%) cases and 25 (45%) controls. Liver and kidney transplant recipients were 28 (58%) and 18 (32%). Median age was 65 years and 54% were female. Risk factors for ESBL EB included liver transplant recipients (p < 0.001), second surgery < 30 days (p=0.01), primary graft dysfunction (p=0.02), and acute rejection < 3 months prior to BSI (p=0.001) (Table 1). Both 30-day (p=0.001) and 90-day (p=0.015) mortality were higher in the ESBL EB group. ST131 (57%) and ST410 (21%) were the predominant sequence types identified in ESBL E. coli isolates. Among ESBL K. pneumoniae, ST16, ST39, ST45, and ST219 were common. Resistome analysis identified β-lactam mutations in all isolates (figure 1 and figure 2). Additional resistance genes that were not phenotypically expressed in ESBL isolates were identified.

**Figure 2:**
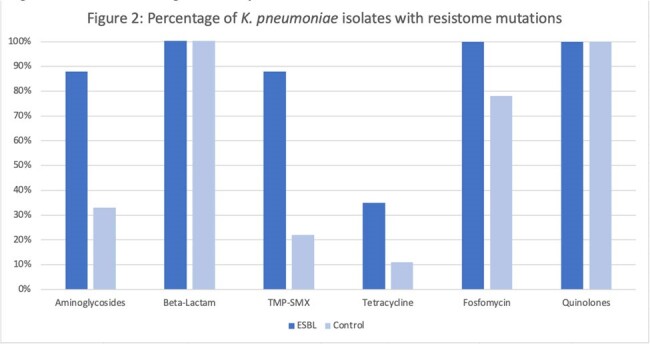
Percentage of K. pneumoniae isolates with resistome mutations

**Conclusion:**

Our study demonstrates the high mortality associated with ESBL EB in SOTr and highlights the risk factors and molecular characteristics of these infections. Clinical significance of the additional resistance genes identified needs further investigation. These findings provide insights for targeted management strategies in this high-risk population.

**Disclosures:**

**Mayur Ramesh, MD**, AstraZeneca: Advisor/Consultant|Moderna: Advisor/Consultant|Pfizer: Advisor/Consultant

